# Folate receptor alpha (FRA) expression in breast cancer: identification of a new molecular subtype and association with triple negative disease

**DOI:** 10.1186/2193-1801-1-22

**Published:** 2012-09-28

**Authors:** Daniel J O’Shannessy, Elizabeth B Somers, Julia Maltzman, Robert Smale, Yao-Shi Fu

**Affiliations:** 1 Department of Diagnostics Development, Morphotek, Inc, 210 Welsh Pool Road, Exton, PA 19341 USA; 2 Department of Clinical Development, Morphotek Inc, 210 Welsh Pool Road, Exton, PA 19341 USA; 3 Laboratory Corporation of America, 2440 South Sepulveda Boulevard, Suite 220, Los Angeles, CA 90064 USA; 4 Quest Diagnostics, 8403 Fallbrook Avenue, West Hills, CA 91304 USA

**Keywords:** Folate receptor alpha, FRA, Breast cancer, Triple negative breast cancer, Immunohistochemistry, IHC

## Abstract

Given that several targeted therapies directed towards folate receptor alpha (FRA) are in late stage clinical development, the sensitive and robust detection of FRA in tissues is of paramount importance relative to patient selection, prognosis and prediction. In the present study we undertook an immunohistochemical evaluation of expression of FRA in breast cancer samples using formalin-fixed, paraffin-embedded (FFPE) tissues, primarily invasive ductal carcinomas, using a newly described monoclonal antibody, 26B3. Samples assessed included both tissue microarrays (TMA) and whole tissue sections from archival tissue blocks. Normal breast shows a highly restricted expression of FRA to luminal membrane staining of secretory ductal cells, consistent with FRA secretion into milk. In early stage (stages I-III) invasive ductal carcinomas, FRA staining was observed in approximately 30% of all samples, independent of molecular subtype (estrogen receptor (ER)/progesterone receptor (PR)/human epidermal growth factor receptor type 2 (Her2)). However, FRA expression was shown to associate with ER/PR negative tumors relative to ER/PR positive tumors (p = 0.012) and perhaps more importantly, with triple negative breast cancers (TNBC; p < 0.0001). FRA immunoreactivity was also shown to be retained in stage IV metastatic breast cancer samples from diverse anatomic sites including lymph node and bone. In metastatic breast cancer, FRA was shown to be expressed in 86% of TNBC patients. Taken together, these data suggest that FRA expressing breast cancer represents a novel molecular subtype and, further, may represent a new therapeutic target for this devastating disease.

## Introduction

According to Global Cancer Facts & Figures 2^nd^ Edition, in 2008 the estimated worldwide new cases for breast cancer were 1,383,500 with a projected 458,400 deaths and a mortality rate of approximately 33%. In the U.S., 229,060 new cases of breast cancer and 39,920 deaths from this disease are expected in 2012 ([Siegel & Naishadham 2012]). Treatment for breast cancer is currently tailored according to cellular protein expression. Estrogen receptor/progesterone receptor (ER/PR) expressing breast cancers are treated with endocrine therapy. The treatment armamentarium of human epidermal growth factor receptor 2 (Her2) overexpressing breast cancers includes an anti-Her2 agent. The triple negative breast cancers (TNBC) that do not express ER, PR or Her2 are treated with traditional cytotoxic chemotherapy alone. New therapeutic approaches for this poor prognosis breast cancer subtype are sorely needed.

Folate, or vitamin B9, is an essential cofactor in the synthesis of purines and pyrimidines and other cellular methylation reactions including DNA, proteins and lipids ([Elnakat & Ratnam 2004]). The folate receptors (folate receptor alpha, FRA; beta, FRB, gamma, FRG; and delta, FRD) constitute a family of proteins that, at least in part, mediate accumulation of folate into cells, regulate folate homeostasis and may have effects on cellular proliferation ([Elnakat & Ratnam 2004];[Kelemen 2006]). FRA, a glycosylphosphatidylinositol (GPI)-anchored cell surface glycoprotein, has a very limited tissue distribution. In normal tissue, FRA is mainly expressed on the apical surface of a subset of polarized epithelial cells including parotid, kidney, lung, thyroid and breast (Weitman et al. [1992a];[Weitman et al. 1992b];[O’ Shannessy et al.2011];[O’Shannessy et al. 2012]). Previous studies have also reported FRA to be expressed on carcinomas of the ovary and endometrium, non-small cell lung adenocarcinoma, clear cell renal carcinoma, colorectal carcinoma, and breast carcinoma ([Weitman et al. 1992a]; [Weitman et al. 1992b]; [O’Shannessy et al. 2011];[O’Shannessy et al. 2012]; [Franklin et al. 1994];[Ross et al. 1994]; [Wu et al. 1999]; [Bueno et al. 2001]; [Parker et al. 2005]; [Shia et al.2008]).

The limited tissue distribution of FRA and its specific expression on certain malignancies makes FRA an attractive target for directed therapies. Indeed, the potential to exploit the differential expression of FRA for targeted cancer therapy has long been appreciated. Two primary approaches have been explored, one involving targeted drug delivery via folate-conjugated therapeutic compounds that binds both FRA and FRB ([Low & Kularatne 2009]; [Dosio et al. 2010]), while another approach involves direct targeting and tumor cell death via humanized anti-FRA monoclonal antibodies ([Ebel et al. 2007]; [Konner et al. 2010]; [Spannuth et al. 2010]). Both approaches have advanced to late-stage clinical development in ovarian cancer.

In order to further support the development of such therapeutic strategies, it is important to identify patients who may benefit from FRA-targeted therapy, particularly in cancers where the frequency and degree of expression is not ubiquitous, such as in breast cancer ([Franklin et al. 1994]; [Shia et al. 2008]; [Stein et al. 1991]). Additionally, reports that FRA expression levels may be associated with disease stage or survival in ovarian cancer or non-small cell lung cancer, suggest that FRA may be a useful prognostic marker ([O’Shannessy et al. 2012]; [Toffoli et al. 1997]; [Toffoli et al. 1998]; [Iwakiri et al. 2008]). Specific and sensitive methods to detect FRA expression in biological samples such as tissue are essential if it is to be pursued as a potential anti-cancer therapy.

Here we describe and define the expression pattern of FRA in breast cancer using a recently reported monoclonal antibody, MAb 26B3 ([O’Shannessy et al. 2011]; [O’Shannessy et al. 2012]), and demonstrate a strong association of FRA expression with TNBC. FRA expressing breast cancers may represent a unique and novel molecular subtype of breast cancer that may be amenable to FRA-targeted therapeutic interventions.

## Materials and methods

### Tissues

#### Commercial tissue microarray (TMA)

The breast cancer TMA [breast invasive ductal carcinomas (catalog # BR1503a; 72 cases, duplicate cores)] was obtained from US Biomax, Inc. (Rockville, MD). Demographic details for this TMA can be found at: http://www.biomax.us/. These samples were predominantly from women under the age of 60 (range = 19-69 years).

#### Whole section FFPE slides

Individual formalin-fixed, paraffin-embedded (FFPE) slides were obtained from the archives of Genzyme Genetics.

No information is available on the treatment regimens for the patients that contributed the samples analyzed in the present study.

### Immunohistochemistry

IHC was performed using FFPE specimens (TMA or whole sections) and a MACH4 Universal HRP-Polymer Detection Kit (Biocare Medical, Concord, CA). FFPE specimens were sectioned at 5 um onto positively-charged glass slides and heated for approximately 60 min at 60°C. Slides were deparaffinized in three sequential baths of xylene for 3 min each, transferred to three sequential baths of 100% alcohol for 3 min each, followed by three sequential baths of 95% alcohol for 3 min each and then rinsed for 5 min in deionized (DI) water. Slides were then pretreated in Diva heat-induced epitope retrieval solution (Biocare Medical) diluted 1:10 in DI water and placed inside a pressurized decloaking chamber already filled with 500 mL of DI water. For antigen retrieval, slides were incubated for 15 min inside the decloaking chamber in which pressurized incubation reaches a maximum of 125°C at 16 PSI for 30 sec and then cooled for 15 min down to 95°C. After cooling to RT, slides were washed in three sequential baths of Tris Buffered Saline/0.1% Tween-20 wash buffer (TBST) for 3 min each and subsequently placed into Peroxidase-1 (Biocare Medical) blocking solution for 5 min at RT. After washing in TBST as above, Background Sniper (Biocare Medical) serum-free universal blocking reagent was applied for 10 min at RT. Slides were then incubated with purified MAb 26B3.F2 ([O’Shannessy et al. 2011]) at 2.5 μg/mL diluted in Antibody Diluent (Dako North America, Inc., Carpinteria, CA) or Universal Negative Control [mouse ready-to-use negative control antibody (Dako, for negative isotype tissue)] for 60 min at RT. After washing, slides were incubated with MACH4 Mouse Probe Primary Antibody Enhancer for 15 min, followed by Polymer-HRP reagent for 20 min, developed with a 3,3'-diaminobenzidine tetrahydrochloride (DAB) solution (Dako) for 5 min and counterstained with hematoxylin (Dako) for 2 min, all incubations being performed at RT. Scoring for staining was performed by a single board-certified pathologist, using customary scoring for intensity and the percent of the tumor stained at each intensity.

### Scoring method

**Figure 1:**
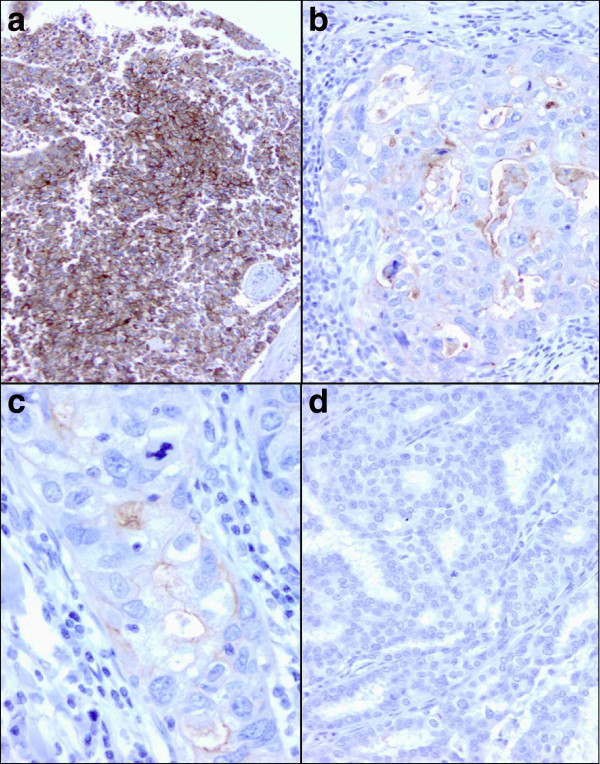
**FRA IHC scoring criteria.a** Strong 3+ membrane staining is clearly visible at 10x. The membrane staining is intense, thick and circumferential (x10). **b** 2+ moderate membrane staining on the luminal borders of malignant cells in a poorly differentiated ductal carcinoma (x20). **c** 1+ weak membrane staining is seen on the luminal border of poorly differentiated ductal carcinoma cells. It requires 40x objective to visualize the thin, incomplete membrane staining (x40). **d** No membrane staining is seen in this well-differentiated ductal carcinoma (x20).

In this study, FRA IHC membrane and intracellular staining intensity was scored as 0, no staining; 1+, weak; 2+, moderate and 3+, strong. The percent of cells staining at each intensity in the sample was also determined. Sections were analyzed under 4x, 10x, 20x and 40x objectives. 3+ strong membrane staining was readily visualized under 4x and confirmed at 10x objective. 2+ moderate membrane staining was visible at 10x and confirmed at 20x, whereas 1+ weak staining required 20x or 40x objectives (Figure 1 a-d). In the presence of 3+ staining, the membrane was thick and occurred at apical and lateral cell borders. In tangential sections, a complete circumferential pattern was evident (Figure 1a). 2+ membrane staining was weaker in intensity and thinner than 3+ membrane staining, usually localized on the apical luminal borders and occasionally on lateral cell borders. 1+ weak membrane staining was generally limited to the luminal borders. The accompanying intracellular staining was variable, depending on the type of tumors.

### Statistical analyses

All statistical analyses were performed using GraphPad Prizm 5 (GraphPad Software Inc., La Jolla, CA).

### Positive staining result and TMA core rejection

A sample (TMA core or FFPE slide) was considered positive for FRA expression if the percentage of the tumor area considered by the reading pathologist (Y-S Fu) to be positive for membranous staining was greater than or equal to 5% at any intensity. A TMA core was rejected and therefore not included in the analyses if the reading pathologist determined it was either missing entirely (empty core), was composed of necrotic tissue or was deemed to represent normal tissue. Histopathologic diagnosis of cores was made by the reading pathologist.

### The M-Score – a semi-quantitative staining algorithm

The M-score (O’Shannessy et al. [2012]), a metric for staining of each sample was defined as follows:

In the equation,  is the percentage of tumor stained at intensity *j* for patient *i* and  is the absolute value of the intensity. The metric has a theoretical range from zero (no positive staining) to 50 (100% 3+). As such, the M-score is a weighted score of FRA IHC tumor cell membrane staining that captures both the proportion of FRA positive cells and staining intensity.

The M-scores for each patient/sample were averaged over duplicate TMA cores, where appropriate. If a determination (core) was void of results, i.e. no tumor present or necrotic tissue, the M-score was assigned to the non-void determinations.

The expression rate for FRA expression was calculated as the proportion of tumors that were stained positive according to the definition of a positive result (≥5% tumor cell membrane staining). This procedure was also applied within specific histology subgroups. Differences for mean values were determined using Fisher’s exact test or one-way ANOVA with *post hoc* tests controlling for overall type I error.

## Results

As previously described ([O’Shannessy et al. 2011]), MAb 26B3 is a unique, high affinity antibody shown to be highly specific for FRA with no cross-reactivity to the other three members of this receptor family, namely FRB (folate receptor beta), FRG (folate receptor gamma) or FRD (folate receptor delta). MAb 26B3 has been shown to recognize FRA on FFPE sections of various normal tissues, including breast ([O’Shannessy et al. 2012]). Importantly, the staining pattern of FRA by MAb 26B3 was consistent with a membranous localization (Figure 1), although diffuse intracellular staining was also observed. In the absence of membrane staining, intracellular staining was rarely present. Intracellular staining for FRA is expected given that the receptor cycles, carrying folates with it, to the intracellular compartment while remaining membrane associated, by an endocytotic mechanism ([Elnakat et al. 2009]).

### FRA expression on the breast cancer TMA

**Figure 2:**
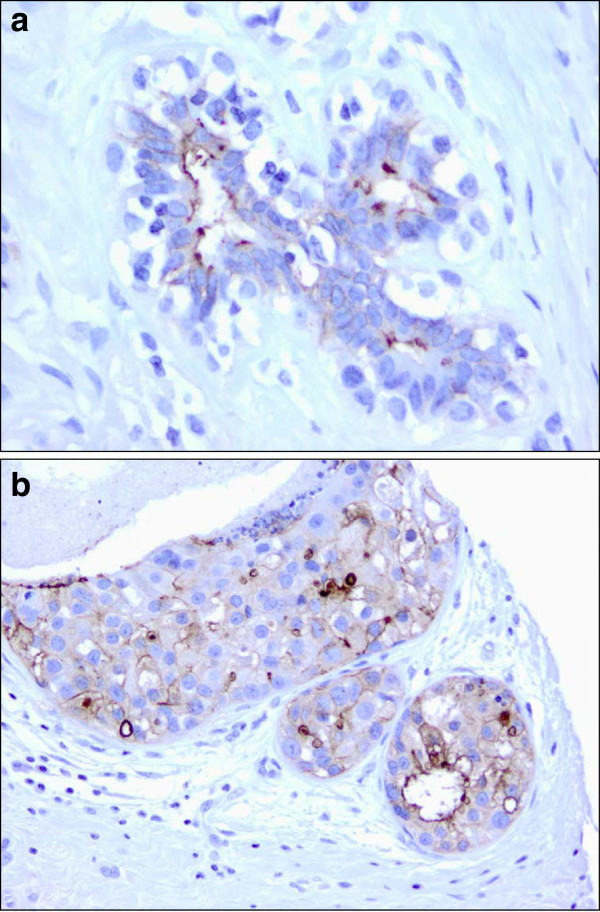
**FRA staining in normalbreast tissue and DCIS.a** Normal breast tissue: strong 3+ membrane staining is seen on the luminal border of secretory cells. Myoepithelial cells in the outer layer of the duct are not stained (x40). **b** Ductal carcinoma in situ of breast, intermediate grade: the majority of tumor cells reveal 3+ strong or 2+ moderate membrane staining on the luminal and lateral cell borders (x20).

The distribution of histologies present on the breast cancer TMA are shown in Table 1, the majority (83%) of the cases represented being identified as invasive ductal carcinoma (IDC). The TMA included two normal breast samples, and as previously described ([O’Shannessy et al. 2011]; [O’Shannessy et al. 2012]), both were positive for FRA expression as determined by MAb 26B3. Membrane staining of normal breast is restricted to the luminal borders of secretory cells while myoepithelial cells in the outer layer of the duct are negative (Figure 2a). The staining of normal breast is not unexpected in that FRA is secreted into breast milk and believed to be a source of bound folates for the developing embryo ([Elnakat & Ratnam 2004]).

**Table 1:** **Distribution of FRA expressionacross breast histologies –TMA data**

Histology	FRA positive	FRA negative	Total
	N (%)	N (%)	
Normal	2 (100%)	0 (0%)	2
Fibroadenoma	2 (67%)	1 (33%)	3
Cystosarcoma	0 (0%)	2 (100%)	2
DCIS – Ductal carcinoma in situ	1 (17%)	5 (83%)	6
ILC – Invasive lobular carcinoma	0 (0%)	1 (100%)	1
IDC – Invasive ductal carcinoma	18 (31%)	41 (69%)	59
Total samples:	21 (30%)	50 (70%)	71

***FRA*** folate receptor alpha, ***TMA*** tissue microarray.

Of the 71 evaluable cases on the TMA, 21 (30%) were shown to be FRA(+) using the criteria of ≥5% of tumor cells exhibiting membrane staining. Two of three fibroadenoma cases (67%), 0/2 cystosarcoma cases (0%) and 1/6 ductal carcinoma in situ (DCIS) cases (17%) were FRA(+) (Figure 2b). The single invasive lobular carcinoma (ILC) was FRA(−). The small number of samples represented by these histologies precludes any definitive statement with respect to FRA expression rates and further work is warranted. However, of the 59 IDC samples 18 (31%) were FRA(+) (Table 2). In this sample set, no significant differences were noted for FRA expression in IDC relative to stage, nodal status or grade (Table 2), although it should be noted that the number of FRA(+) samples in this analysis is relatively small.

**Table 2:** **IDC molecular subtype analysisrelative to FRA staining– TMA data**

Variable	FRA positive	FRA negative	Total	P value^a^
	N (%)	N (%)		
Marker				
ER/PR(+)	4 (14%)	24 (86%)	28	
ER/PR(-)	14 (45%)	17 (55%)	31	0.012
Her2(+)	2 (15%)	11 (85%)	13	
Her2(-)	16 (35%)	30 (65%)	46	0.307
ER/PR/Her2(-)	12 (67%)	6 (33%)	18	<0.0001
				[ER/PR(+) or Her2(+) *vs* ER/PR/Her2(-)]
TNM Classification				
T1	3 (43%)	4 (57%)	7	
T2	10 (26%)	29 (74%)	39	
T3	5 (63%)	3 (37%)	8	
T4	0 (0%)	5 (100%)	5	
Nodal Status				
N0	18 (35%)	33 (65%)	51	
N1/N2^b^	0 (0%)	8 (100%)	8	0.092
Tumor Grade				
Grade 1	1 (14%)	6 (86%)	7	
Grade 2	12 (36%)	21 (64%)	33	0.393
Grade 3	5 (26%)	14 (74%)	19	0.6465^c^

***IDC*** invasive ductal carcinoma, ***TMA*** tissue microarray, ***FRA*** folate receptor alpha, ***ER*** estrogen receptor, ***PR*** progesterone receptor, ***Her2*** human epidermal growth factor receptor type 2, ***TNM*** tumor node metastasis.

^a^ P values calculated via 2X2 contingency table analysis using Fisher’s exact test.

^b^ 4/8 (50%) of N1/N2 samples were Her2(+).

^c^ Grade 1 *vs* Grade 3.

**Figure 3:**
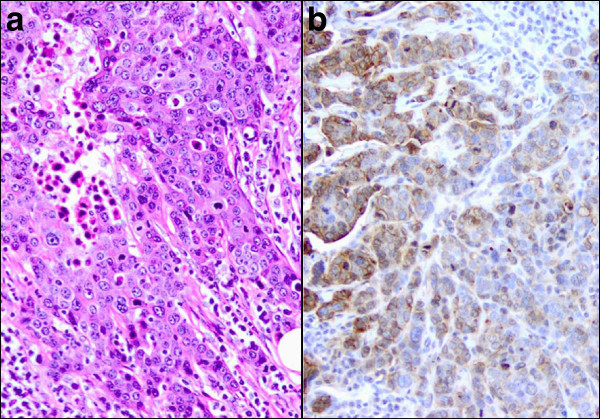
**Triple negative [ER/PR/Her2(−)] poorlydifferentiated ductal carcinoma.a** Tumor cells are arranged in solid nests and present with high nuclear grade, prominent nucleoli and active mitosis. Cell borders are ill-defined. There are scattered apoptotic cells with pyknotic nuclei and densely eosinophilic cytoplasm (x20). **b** In the immunohistochemical stain for FRA, about 60% of tumor cells demonstrate membrane staining ranging from 1+ to 3+ (x20).

Of the 18 FRA(+) IDC cases, the majority (89%) were Her2(−) suggesting that FRA expression is negatively correlated with Her2 expression in breast cancer (Table 2). Further, of the 18 FRA(+) IDC cases there was a statistically significant difference between ER/PR(+) and ER/PR(−) cases (p = 0.012; Table 2). In addition, of the 4 FRA(+) samples that were also ER/PR(+), all were Her2(−). A statistically significant difference was seen for FRA(+) samples relative to expression of either ER, PR or Her2 versus ER/PR/Her2(−), or triple negative disease (p < 0.0001). Sixty-seven percent of the 18 TNBC specimens express FRA (Figure 3). Taken together, these data support the claim that FRA expression is enriched in the TNBC subtype and may represent a novel molecular subtype of breast cancer.

### Analysis of individual slides from Her2(−) metastatic breast cancer patients

The TMA described above was composed primarily of early stage breast cancers: stage I, 6/60 (10%); stage II, 44/60 (73%); stage III, 10/60 (17%); no metastatic breast cancer cases were represented on the TMA (Table 3). Therefore, to confirm and extend the results obtained on the TMA, we identified 61 archival FFPE tissue blocks from stage IV (T4) Her2(−) breast cancers with known ER/PR expression ranging from 0-100% positive. Importantly, all 61 of these samples were from metastases, not the primary tumor.

**Table 3:** **Distribution of FRA expressionrelative to hormone receptorstatus and tumor gradein a Her2(−) metastaticbreast cancer cohort**^**a**^

Variable	FRA positive	FRA negative	Total	P value^b^
	N (%)	N (%)		
Marker				
ER/PR(+)	3 (14%)	20 (86%)	23	
ER/PR/Her2(−)	19 (50%)	19 (50%)	38	0.0054
				[ER/PR(+) vs ER/PR/Her2(-)]
Tumor Grade				
Grade 1	3 (30%)	7 (70%)	10	
Grade 2	11 (28%)	28 (72%)	39	1.0
				(Grade 1 vs Grade 2)
Grade 3	8 (67%)	4 (33%)	12	0.037
				(Grade 1 or 2 vs Grade 3)
Total Samples	22 (36%)	39 (64%)	61	

**FRA** folate receptor alpha, **Her2** human epidermal growth factor receptor type 2, **ER** estrogen receptor, **PR** progesterone receptor.

^a^ All samples were whole tissue FFPE (formalin-fixed, paraffin-embedded) slides.

^b^ P values calculated via 2X2 contingency table analysis using Fisher’s exact test.

FRA expression was found in 22/61 (36%) of these patients, demonstrating that the percent of FRA(+) specimens/tumors determined in early stage disease is retained in late stage metastatic disease, at least in a Her2(−) population (TMA expression rate = 35%; stage IV metastatic disease = 36%). Of the 22 FRA(+) stage IV metastatic cases, only three (14%) showed any expression level for ER/PR which tended to be in the low range (up to 30%). As such, 19/22 (86%) FRA(+) patients were of the TNBC molecular subtype. As with the data obtained in early stage disease on the TMA, triple negative samples are overrepresented in the FRA(+) population. However, both data sets support the assertion that FRA staining associated more strongly with triple negative disease. In metastatic disease, there was a significant difference between FRA(+) samples that were also ER/PR(+) or ER/PR(−) (p = 0.0054), as seen on the TMA, but also a significant difference in FRA expression by grade of disease (grade 1 or grade 2 versus grade 3, p = 0.037).

**Figure 4:**
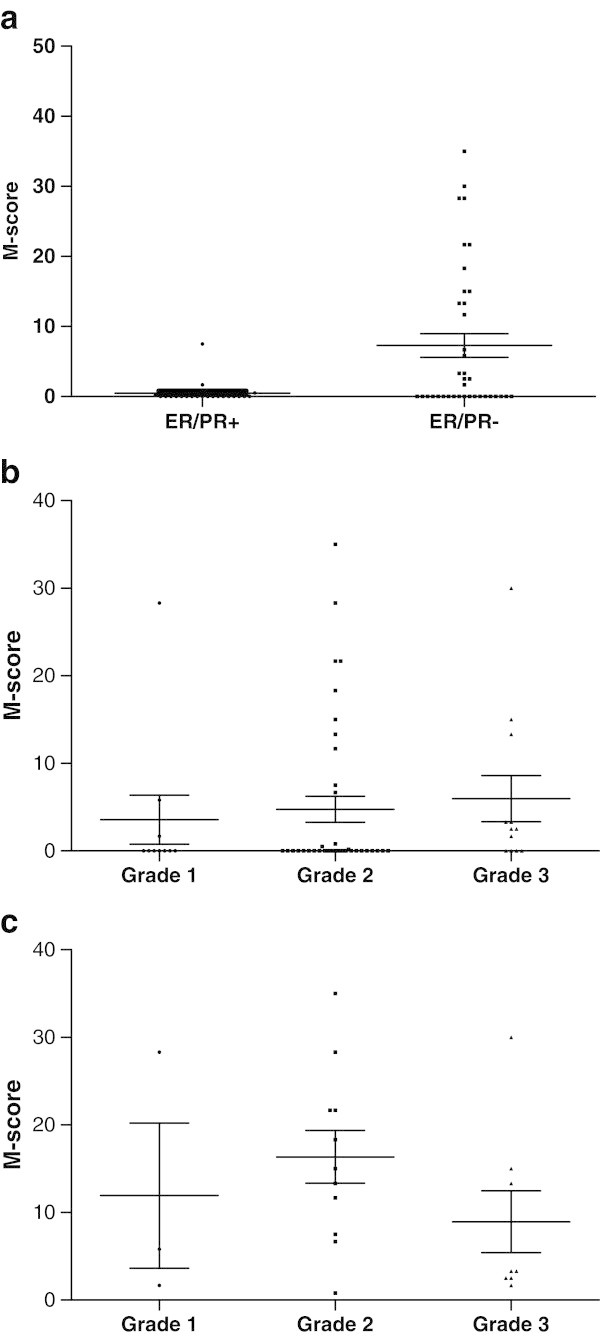
**M-score Distribution of FRAExpression in Her2(−) MetastaticBreast Cancer. a** Distribution of the level of FRA expression in 61 Her2(−) metastatic breast cancer samples based on molecular subtype: ER/PR(+) versus ER/PR(−) p = 0.0029 (two-tailed *t*-test). **b** Distribution of the level of FRA expression based on grade across 61 Her2(−) metastatic breast cancer samples. The differences in the means of the M-scores were not significant (one-way ANOVA). **c** Distribution of the level of expression of FRA across grade in FRA positive (n = 22) metastaic breast cancer samples. The differences in the means of the M-scores were not significant (one-way ANOVA).

The semi-quantitative M-score ([O’Shannessy et al. 2012]) was also used to analyze the pattern of FRA staining (intensity and percent of tumor cells) of metastatic breast cancer samples. While a significant difference could be demonstrated between the ER/PR(+) versus the ER/PR(−) populations (Figure 4a; p = 0.0029), no such difference was evident for grade of disease either across the entire population (Figure 4b) or within the FRA(+) population (Figure 4c). These data are consistent not only with FRA expression overall, but also with the data described for the early stage TMA samples. Taken together, the present data demonstrate a strong association between FRA expression and TNBC and further support the notion that FRA(+) breast cancer may represent a new molecular subtype of this disease.

**Figure 5:**
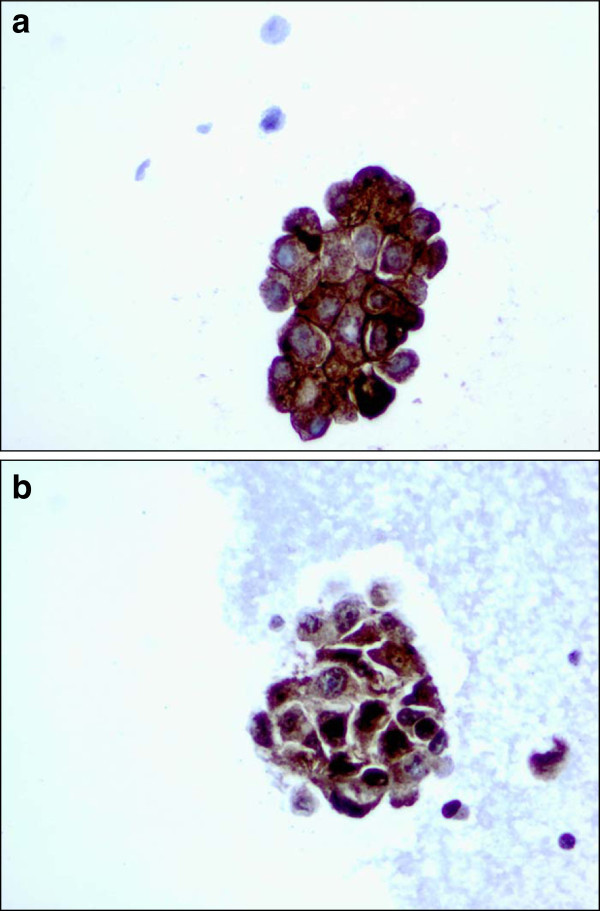
**Use of breast cancerFNAs for FRA IHC.a** CTBA08494A (40X) – A cluster of malignant cells with 3+ membranous staining and 3+ intracellular staining. **b** CTBA08496A (40X) – A cluster of malignant cells with mostly 3+ intracellular staining.

The samples represented in the FFPE blocks from stage IV metastatic disease were obtained from a number of metastatic sites including lymph node, bone, skin and liver, as well as fluid and fine needle aspirate (FNA) samples obtained primarily from pleura and paracentesis. Several of these ‘fluid biopsies’ were stained positive for FRA (Figure 5) suggesting the general applicability of the described IHC methodology to multiple samples types. However, given the small sample numbers in the present study, additional work on the suitability of FNAs as a FRA IHC diagnostic sample source are warranted. It should be noted, however, that FNAs have also been demonstrated to be positive for FRA expression by IHC in non-small cell lung adenocarcinoma ([O’Shannessy et al. 2012]).

## Discussion

Currently, breast cancer is grouped into three subtypes that dictate therapy: ER and/or PR expressing, Her2 expressing, or TNBC which implies the absence of these three receptors. Hormone responsive breast tumors represent approximately two-thirds of all breast cancers ([Bauer et al. 2007]). Treatment at some point during the course of therapy will necessarily include hormonal agents. Endocrine therapy has been shown to both prolong life and decrease treatment related morbidity. Her2 overexpressing breast cancer represents approximately 15-20% of breast cancers ([Bauer et al. 2007]). Although treatment with trastuzumab has vastly improved survival in patients with Her2 positive tumors, the prognosis for this group remains worse than for their hormone receptor expressing counterparts. The balance of breast cancers lack all three of these receptors and are referred to as triple negative.

It is unclear from the current investigation if the FRA(+) subset represents a particular TNBC histotype. It is conceivable that the FRA expressing cancers may fall into the basal-like category that are also known to sometimes be BRCA(+), characterized high grade infiltrating ductal carcinomas that can show necrosis ([Metzger-Filho et al. 2012]), or by contrast the luminal androgen receptor subtype that often present with bone and lymph node involvement but are more indolent by nature ([Gelmon et al. 2012]).

There are no currently available targeted therapies for TNBC and their prognosis remains decidedly worse than those with hormone receptor expression. Recently, PARP inhibitors were assessed in this TNBC patient population with the hope of improving survival and outcome. However, a phase III randomized clinical trial failed to show benefit ([O’Shaughnessy et al. 2011]). Treatment for this molecular subtype of breast cancer remains untargeted cytotoxics.

FRA is an interesting and relevant biomarker in cancer therapy. This GPI-anchored protein at least in part serves to bind and transport folate, primarily 5-methyltetrahydrofolate, the predominant plasma folate, into cells. However, its expression is not necessary for cytosolic folate accumulation and purine and pyrimidine synthesis as there are multiple portals of folate entry, primarily the reduced folate carrier (RFC), ubiquitous on all mammalian cells ([Elnakat & Ratnam 2004]). Hence, blocking or disrupting FRA will not a priori deprive a cell of necessary folate and kill it. However, FRA has been reported to impart a growth advantage to cells expressing the receptor, especially in low folate environments ([Luhrs et al. 1992]). Furthermore, FRA’s high expression on epithelial malignancies, compared to normal tissues, provides the rationale for its use as a potential targeted cancer therapy. FRA targeted therapies are currently in clinical investigation in both ovarian and lung cancers and it is interesting to speculate on the potential value of such an approach in FRA expressing breast cancer. It is worth repeating that FRA is expressed in normal breast, restricted to the luminal borders of secretory cells, consistent with the secretion of FRA into breast milk. No other breast cell type has been shown to express FRA. Such an expression pattern in normal breast tissue was seen in the present study although it should be noted that not all samples, especially TMA cores, contain normal tissue. The expression of FRA in carcinomas may reflect the cellular origin of the carcinoma and may, at least in part, explain the approximately 30% incidence in IDC demonstrated here.

Hormone receptor positive tumors appear to have a lower incidence of FRA expression. FRA expression has been shown to be regulated by steroid hormones, particularly estrogens ([Rochman et al. 1985]; [Kelley et al. 2003]). Specifically, 17β-estradiol has been demonstrated to down-regulate FRA expression by direct action of the estrogen receptor on the P4 promoter of FRA suggesting a negative correlation between the expression of ER and FRA. The data presented herein is in line with these findings in that there was a clear association between FRA(+) and ER(−) samples, i.e. ER(−) samples were significantly more likely to be FRA(+).

A recent report ([Hartmann et al. 2007]) on a cohort of 63 invasive breast cancers, roughly equally distributed between good and poor outcome, demonstrated that strong FRA staining was highly associated with poor outcome. While the authors did not report on Her2 status of these patients, it is interesting to speculate that the poor outcome group may have included a significant TNBC population. Studies are underway to assess the prognostic significance of FRA expression in breast cancer and any relationship to molecular subtype and histotype.

Our data, although limited by a small sample size, demonstrate that approximately 30% of breast cancers express FRA and suggest that as many as 70–80% of stage IV metastatic TNBC tumors express this receptor. FRA expressing breast cancer may, therefore, represent an important and clinically significant subset of breast cancer and in particular triple negative disease. FRA targeted therapies, alone or in combination with cytotoxics, may represent a novel approach to treatment for this disease with a high unmet medical need.

Further work is clearly needed to confirm and extend the present findings and to provide a clinical correlation with FRA expression. Additional correlations with disease progression and response to treatment would be interesting and potentially valuable in disease monitoring and response assessments.

## Abbreviations

FRA: Folate receptor alpha, folate receptor α
FRB: Folate receptor beta, folate receptor β
FRG: Folate receptor gamma, folate receptor γ
FRD: Folate receptor delta, folate receptor δ
IHC: Immunohistochemistry
MAb: Monoclonal antibody
FFPE: Formalin-fixed, paraffin-embedded
FNA: Fine needle aspirate
TMA: Tissue microarray
IDC: Invasive ductal carcinoma
DCIS: Ductal carcinoma in situ
ER: Estrogen receptor
PR: Progesterone receptor
Her2: Human epidermal growth factor receptor type 2
TNBC: Triple negative breast cancer
TNM: Tumor node metastasis.
